# Supplementary material for: A high-resolution mRNA expression time course of embryonic development in zebrafish
Source: eLife. 2017 Nov 16;6:e30860. doi: 10.7554/eLife.30860 (PMC5690287; doi:10.7554/eLife.30860)
Supplement: Supplementary file 6. [file elife-30860-supp6.zip › biolayout-clusters-files/Cluster211-genes.html]

Cluster211


# Cluster211: Genes

| Ensembl ID | Gene Name | Chr | Start | End | Biotype |
| --- | --- | --- | --- | --- | --- |
| ENSDARG00000079575 | aff3 | 9 | 21679953 | 21715787 | protein\_coding |
| ENSDARG00000038489 | b3gnt7 | 22 | 36687671 | 36692452 | protein\_coding |
| ENSDARG00000060526 | bmp3 | 5 | 39067119 | 39073324 | protein\_coding |
| ENSDARG00000076440 | btbd6a | 13 | 33263666 | 33268285 | protein\_coding |
| ENSDARG00000017121 | mafba | 23 | 3462556 | 3465717 | protein\_coding |
| ENSDARG00000012968 | rhoub | 15 | 9014506 | 9026086 | protein\_coding |
| ENSDARG00000019930 | tal1 | 22 | 16509305 | 16519060 | protein\_coding |
